# Emotional exhaustion and psychological well-being as predictors of moral disengagement in nursing students: a cross-sectional study

**DOI:** 10.1186/s12909-026-10155-4

**Published:** 2026-08-13

**Authors:** Mohammad Reza Sheikhi, Mohammad Ali Soleimani, Zainab Alimoradi, Haniyeh Shadin, Mohammadreza Asadi

**Affiliations:** 1 Social Determinants of Health Research Center, Research Institute for Prevention of Non-Communicable Diseases, Qazvin University of Medical Sciences, Qazvin, Iran; 2 Student Research Committee, Qazvin University of Medical Sciences, Qazvin, Iran

**Keywords:** Moral disengagement, Psychological well-being, Emotional exhaustion, Nursing students, Predictors

## Abstract

**Background and aims:**

Nursing students, as the future architects of healthcare delivery, routinely navigate ethically complex clinical environments that render them susceptible to moral disengagement. This self-regulatory cognitive process decouples internal moral standards from conduct, enabling individuals to rationalize unethical behavior without experiencing self condemnation. Despite its profound implications for patient safety and professional identity formation, the concurrent contributions of erosive occupational factors such as emotional exhaustion and salutogenic personal resources such as psychological well-being remain insufficiently explored in this population. This study therefore sought to disentangle the predictive roles of psychological well-being and emotional exhaustion in moral disengagement among senior nursing students.

**Methods:**

This descriptive cross-sectional study was conducted in 2025 among 203 final year nursing students recruited through census sampling from Qazvin University of Medical Sciences in northwestern Iran. Participants completed a demographic information checklist alongside three validated instruments: Fida’s Moral Disengagement Scale, Rutherford’s Emotional Exhaustion Scale, and Ryff’s Scales of Psychological well-being. Given the nested structure of the data (four moral disengagement subscales per student), data were analyzed using Generalized Estimating Equations (GEE) with robust (Huber‑White) standard errors to account for within‑subject correlations, with the exchangeable working correlation structure selected as the optimal fit following comparison with alternative structures.

**Results:**

The mean scores for moral disengagement, emotional exhaustion, and psychological well‑being were 44.87 (SD = 12.29), 12.17 (SD = 3.98), and 69.12 (SD = 9.73), respectively. The GEE analysis revealed that emotional exhaustion (β = 0.461, *p* < 0.001) and male gender (β = 0.236, *p* < 0.001) emerged as the strongest positive predictors of moral disengagement. Personal growth demonstrated a significant protective effect (β = -0.195, *p* = 0.005), while purpose in life showed a protective trend (β = -0.143, *p* = 0.054). Other dimensions of psychological well-being, including autonomy, environmental mastery, positive relations with others, and self-acceptance, did not show significant independent effects. A significant interaction was observed between emotional exhaustion and moral disengagement subscales (Wald χ² = 52.356, *p* < 0.001), indicating that emotional exhaustion had the strongest effect on agency distortion (Β = 0.494, *p* < 0.001). The model demonstrated excellent fit (Omnibus test: χ² = 99.183, *p* < 0.001; QIC = 7866.058).

**Conclusion:**

Emotional exhaustion constitutes a potent risk factor for moral disengagement, particularly through the agency distortion mechanism, while personal growth serves as a significant protective buffer. Educational and organizational interventions that systematically target emotional exhaustion while nurturing personal growth and a coherent sense of purpose may substantially mitigate moral disengagement among nursing students, ultimately fostering a more ethically resilient future workforce.

## Highlights

- Emotional exhaustion emerged as the single strongest predictor of moral disengagement among nursing students in the multivariable model.
- Male gender was consistently associated with elevated moral disengagement scores across all measured dimensions.
- Personal growth and purpose in life, two core facets of psychological well-being, exerted significant protective effects against moral disengagement.
- Agency distortion, defined as the external displacement of personal responsibility, was identified as the most prevalent moral disengagement mechanism.
- The comprehensive regression model explained 36.1% of the total variance in moral disengagement, highlighting the critical interplay of affective depletion and psychological resources.

## Introduction

Clinical nursing education environments are arenas saturated with ethical tension. As the future workforce of the healthcare system, nursing students not only advance their practical competencies, professional autonomy, and clinical decision-making through intensive clinical training [1, 2] but are also continually confronted with situations that fundamentally challenge their moral values. Delivering safe, high-quality care depends, above all, on possessing a robust “ethical infrastructure” composed of unwavering commitment to professional principles and sound psychological health [3, 4]. Yet this very infrastructure becomes acutely vulnerable under the weight of hierarchical pressures, relentless clinical supervision, and recurrent ethical conflicts [5, 6]. One of the most insidious and simultaneously most damaging threats to this ethical foundation is the cognitive phenomenon of moral disengagement. This mechanism permits individuals, through a series of mental distortions, to reconstruct and justify behaviors that violate their own ethical standards, thereby freeing themselves from the psychological tension of moral dissonance. For nursing students, who stand at the critical juncture of professional identity formation and the internalization of ethical values, this mechanism represents far more than a transient defense; it constitutes a fundamental threat to the very formation of their future professional conscience [3].

Moral disengagement originates in Albert Bandura’s social cognitive theory and is defined as a faulty self-regulatory mechanism through which individuals selectively deactivate their moral inhibitory processes [7, 8]. Bandura delineated eight specific mechanisms for this process, organized within four primary clusters: behavioral distortion (moral justification, advantageous comparison, euphemistic labeling), agency distortion (displacement and diffusion of responsibility), consequence distortion (minimizing or disregarding outcomes), and victim dehumanization (dehumanization and attribution of blame). In clinical contexts, where hierarchical structures and the distribution of responsibility are inherently complex, agency distortion mechanisms, particularly the displacement of responsibility onto external factors such as workload volume or physicians’ decisions, are more prevalent [9]. Consider, for example, a student who, due to time constraints, delivers incomplete patient care. The student may invoke responsibility displacement, attributing fault to excessive workload and staffing shortages, or else employ moral justification, sanitizing the action with the reasoning that “under these very conditions, the patient was still being monitored.” This process, by temporarily disabling moral self sanctions, allows the individual to perpetrate behavior that contravenes professional standards without experiencing guilt or shame [8–10]. The consequences of this mechanism are traceable at two distinct levels: systemically, moral disengagement is linked to the erosion of professional accountability, a rise in counterproductive work behaviors, and a tangible decline in the quality of care [11]; individually, the mechanism can intensify psychological distress, depression, and emotional exhaustion, generating a vicious cycle of moral and psychological depletion [12]. This brings a fundamental question to the fore: what specific factors render nursing students particularly susceptible to this form of moral vulnerability?

A growing body of evidence indicates that emotional exhaustion, as the central dimension and frequently the initial stage of occupational burnout, plays a pivotal role in depleting self-regulatory resources [13]. Emotional exhaustion is defined as a state of psychological depletion and the complete drainage of emotional energy reserves [14, 15]. Nursing students, owing to their constant exposure to patient suffering, academic pressures, the expectations of clinical instructors, and the fear of committing clinical errors, are uniquely susceptible to this phenomenon [16, 17]. The clinical training environment routinely exposes these students to high-stress scenarios that elevate clinical anxiety, particularly during initial clinical placements or specialized rotations such as pediatric care, where targeted anxiety-reduction strategies are often warranted [18, 19]. This vulnerability to severe psychological stress and emotional depletion can be further compounded in educational settings characterized by systemic difficulties or political violence [20]. Alarming statistics from across the globe reveal the depth of this crisis; for instance, in a study conducted in China, 45.4% of nursing students expressed no intention to remain in the profession [21], and multiple studies in Iran similarly report a high prevalence of academic and emotional burnout within this population. Far from being a transient experience, emotional exhaustion exerts a systematic impact on physical and mental health and is associated with outcomes including sleep disturbances, depression, diminished motivation, and occupational avoidance behaviors [22, 23].

To understand the mechanistic link between emotional exhaustion and moral disengagement, Hobfoll’s Conservation of Resources (COR) theory provides a powerful explanatory framework [24]. According to this theory, individuals strive to preserve and cultivate their valued resources, whether emotional, cognitive, or physical. Emotional exhaustion is the product of the continuous erosion of these vital resources without adequate replenishment. The critical point here is that moral self regulation, defined as the capacity to inhibit behaviors incompatible with one’s internalized values, is itself an intensely resource-intensive process. It demands the expenditure of substantial cognitive and emotional energy on self monitoring, the suppression of unethical impulses, and the endurance of tension arising from moral dissonance. When an individual’s psychological resources are drained by emotional exhaustion, their capacity to engage in this costly process is weakened. Under such conditions, the individual unconsciously seeks lower cost coping strategies. Moral disengagement serves precisely as such an evolutionarily shaped cognitive shortcut, one that permits the individual to reduce the psychological tension caused by an ethical conflict not by expending energy on resolving the conflict itself, but merely by reconstructing the situation [9]. Although prior studies, predominantly among employed nurses, have supported this association [25, 26], the dynamics of this mechanism among students, who possess more constrained coping resources and a more fragile professional identity, remain largely unexplored.

The path toward moral erosion is, however, not inevitable. Alongside erosive factors such as emotional exhaustion, positive psychological resources can serve as “protective shields,” immunizing the individual against this vicious cycle. Among these, psychological well-being holds a distinct position. In contrast to hedonic definitions equating well being with happiness and satisfaction, Ryff’s (1989) six-dimensional model adopts a eudaimonic approach, defining psychological well-being as the full actualization of an individual’s potential across the dimensions of self acceptance, positive relations with others, autonomy, environmental mastery, purpose in life, and personal growth [27]. Nevertheless, evidence indicates that this psychological capital is fragile among nursing students; studies have reported that approximately half of these students experience low levels of psychological well-being, a condition that deteriorates with advancing academic years under the mounting pressures of educational and clinical stress [28, 29]. Within this challenging context, contemporary evidence highlights that cultivating personal resilience is fundamentally intertwined with the maintenance of students’ psychological well-being, serving as a prerequisite for successful professional socialization [30].

To illuminate the protective role of psychological well-being against moral disengagement, a complementary theoretical lens proves useful: the developmental roots of this concept reside in Bowlby’s (1969) attachment theory [31]. Just as secure attachment in childhood furnishes an external “secure base” that permits the child to explore and cope with stress, psychological well-being in adulthood functions as an internalized secure base. An individual possessing high levels of psychological well-being, particularly in the dimensions of personal growth (a sense of continued flourishing) and purpose in life (a clear and meaningful direction), is equipped with sufficient internal resources to confront ethical challenges without resorting to destructive mechanisms such as moral disengagement. Effectively, these dimensions help the individual to interpret ethical conflicts not as threats to the self but as opportunities for growth and the consolidation of professional identity. This theoretical lens demonstrates that psychological well-being is not merely a positive emotional state but a foundational self-regulatory resource, one that, in accordance with the buffering hypothesis, can moderate and neutralize the detrimental effects of acute stressors like emotional exhaustion [32]. Thus, alongside emotional exhaustion as a risk factor, psychological well-being may play a decisive protective role in predicting moral disengagement.

Despite this strong theoretical foundation, the research literature suffers from three notable gaps. First, the vast majority of existing studies on moral disengagement and emotional exhaustion have concentrated on practicing nurses, while nursing students, a population at the most sensitive stage of professional identity formation, have rarely been examined [25, 26]. This research neglect is particularly significant because preventive interventions implemented during the student period are far more effective and less costly than attempts to modify entrenched behavioral patterns during professional employment. Second, existing studies have predominantly adopted a unidimensional approach, focusing either on risk factors such as burnout or on protective factors such as resilience. What has remained overlooked is the concurrent and competitive examination of these two sets of factors within a single model; stated differently, it remains unclear which force, erosive or protective, holds the dominant share in predicting moral disengagement. Third, the specific cultural context and educational system of Iran, characterized by a combination of intense academic pressures, familial expectations, constrained educational and clinical resources, and distinct power hierarchies, may generate a unique pattern of these relationships that is not necessarily generalizable from international studies.

To bridge these gaps, the present study, for the first time in Iran, tests a competitive dual domain model in which emotional exhaustion, as an erosive independent variable, and the dimensions of psychological well-being, specifically personal growth and purpose in life, as protective variables, are simultaneously entered into the equation to predict moral disengagement among final year nursing students. The primary innovation of this study lies not in the isolated examination of each variable but in their concurrent analysis and the determination of their relative weight in predicting a cognitive mechanism. This approach provides a more precise insight into the dynamic interplay between erosion and protection within the moral conscience of students. The findings of this study will not only lead to a deeper theoretical understanding of the mechanisms of moral self deception during the early stages of professional identity formation but will also chart a practical, evidence-based roadmap for designing targeted educational interventions, such as programs for managing emotional exhaustion and strengthening personal growth and purpose in life, within the nursing curriculum. Such interventions hold the potential to cultivate a future generation of nurses endowed with high moral resilience. Accordingly, the present study was designed to determine the predictive roles of psychological well-being and emotional exhaustion in moral disengagement among nursing students. The following hypotheses were tested: (1) psychological well-being dimensions are associated with moral disengagement; (2) emotional exhaustion is associated with moral disengagement; and (3) demographic variables are associated with moral disengagement.

## Materials and methods

### Study design and setting

The present study employed a cross secttional design and was conducted cross sectionally in 2025. The target population comprised final year nursing students enrolled at Qazvin University of Medical Sciences, located in northwestern Iran. The research was carried out across multiple teaching hospitals affiliated with the university.

### Participants and sampling

The study population specifically consisted of all undergraduate nursing students in their seventh and eighth academic semesters who were concurrently completing their clinical internship rotations. In the baccalaureate nursing curriculum of the Iranian educational system, final year students undertake clinical internships in hospital settings without the constant, direct supervision of a clinical instructor. This structure grants them considerably greater autonomy in clinical activities and is specifically designed to prepare them for entry into professional nursing practice. Given that these students possess the experience closest to that of practicing nurses, they were deliberately selected as the population of interest for this investigation. Sampling was carried out between January and October 2025 using a census method, also known as total population sampling.

Due to the finite and accessible nature of the target population within the research setting, all final year undergraduate nursing students who met the predefined inclusion criteria were invited to participate. Out of a total of 222 eligible students, 203 were enrolled in the study via the census approach, yielding a high response rate of 91.44%. The inclusion criteria were as follows: (1) enrollment in the seventh or eighth semester of the baccalaureate nursing program (2), successful completion of all prior mandatory clinical courses, and (3) a stated willingness to voluntarily participate in the study and complete the questionnaires. The exclusion criteria comprised: (1) a self reported history of formally diagnosed severe psychiatric disorders or the current use of psychotropic medications (2), incomplete questionnaire responses, defined as leaving more than 20% of the items unanswered, and (3) a history of formal employment in clinical environments, such as working as a nursing assistant or auxiliary nurse, prior to commencing nursing education. The questionnaires were administered to participants individually and in a physical paper based format by the research team. On average, completing the full battery of instruments required approximately 15 min.

### Sample size and power analysis

Although a census method was employed to capture the entire accessible population, the adequacy of the final sample size for the intended statistical analyses was rigorously verified. For the primary analysis, a multivariable linear regression model was planned. Following the widely accepted rule of thumb requiring 10 to 15 participants per predictor variable to ensure model stability, and anticipating up to 10 potential predictor variables (the demographic variables, emotional exhaustion, and the six dimensions of psychological well-being), the minimum required sample size was estimated to be between 100 and 150 participants. A post hoc power analysis was subsequently performed using G*Power software (version 3.1) for a linear multiple regression with 10 predictors, an observed effect size (f²) of 0.56 derived from the study’s final R² of 0.361, and an alpha level of 0.05. The achieved statistical power with the final sample of 203 participants was 1.00, confirming that the study was sufficiently powered for the multivariable analyses conducted.

### Data collection instruments

Data were collected using a structured, four-part instrument package, as detailed below.

#### Part 1: Demographic information checklist

This researcher developed checklist captured essential participant characteristics, including age, gender, academic semester, place of residence, marital status, and the frequency of voluntary activity during the study period (categorized as often, sometimes, or rarely). Additionally, the checklist assessed the manner in which the participant had initially chosen the nursing discipline and their stated intention to pursue employment in nursing after graduation (categorized as willing, undecided, or unwilling).

#### Part 2: Fida’s moral disengagement scale

Moral disengagement was measured using the scale developed by Fida and colleagues in 2015 [33]. This instrument consists of 22 items, each scored on a five-point Likert scale ranging from “strongly disagree” [1] to “strongly agree” [5]. The total possible score ranges from 22 to 110, with higher scores reflecting a greater propensity for moral disengagement. The scale encompasses four distinct subscales that mirror Bandura’s theoretical clusters: behavioral distortion (7 items), agency distortion (9 items), consequence distortion (3 items), and dehumanization (3 items). The content and construct validity of the original scale were established by its developers, and its reliability was confirmed with a Cronbach’s alpha of 0.86 (Fida et al., 2016). The psychometric properties of the Persian version were rigorously evaluated in an Iranian context by Mohammadpour and colleagues (2023), who confirmed its validity and reported a Cronbach’s alpha of 0.90 [8]. In the present study, the internal consistency of the scale was excellent, as evidenced by a Cronbach’s alpha of 0.91.

#### Part 3: Rutherford’s emotional exhaustion scale

Emotional exhaustion was assessed using the scale developed by Rutherford and colleagues in 2009. This brief, four item instrument is specifically designed to capture feelings of emotional depletion. Each item is rated on a five-point Likert scale with the following anchors: strongly disagree [1], disagree [2], neutral [3], agree [4], and strongly agree [5]. The total scores range from 4 to 20, with a higher score indicating a higher level of emotional exhaustion. The psychometric properties of the Persian adaptation were confirmed in an Iranian sample by Heydari and colleagues (2018). Following their validation process, the instrument’s validity was established, and its reliability was demonstrated with a Cronbach’s alpha of 0.88 [34]. In the current investigation, the scale exhibited good internal consistency, with a Cronbach’s alpha of 0.84.

#### Part 4: Ryff’s scales of psychological well-being

Psychological well-being was measured using the 18 item version of Ryff’s Scales of Psychological well-being [27]. This validated instrument assesses well being across six theoretically derived dimensions: autonomy (items 9, 12, and 18), environmental mastery (items 1, 4, and 6), personal growth (items 7, 15, and 17), positive relations with others (items 3, 11, and 13), purpose in life (items 5, 14, and 16), and self acceptance (items 2, 8, and 10). Responses are recorded on a six-point Likert scale ranging from “strongly disagree” [1] to “strongly agree” [6]. Items 1, 3, 4, 5, 9, 10, 13, and 17 are reverse scored. The total scale score can range from 18 to 108, with higher scores denoting a higher degree of psychological well-being. The original scale developer reported internal consistency coefficients for the subscales ranging from 0.70 to 0.89 [27]. The psychometric properties of the Persian version were thoroughly examined by Khanjani and colleagues (2014). Their confirmatory factor analysis indicated a good model fit for the six factor structure, and the overall internal consistency, as measured by Cronbach’s alpha, was 0.71 [35]. In the present study, the total scale demonstrated an acceptable level of internal consistency, with a Cronbach’s alpha of 0.75.

### Ethical considerations

The present study was reviewed and formally approved by the Ethics Committee of Qazvin University of Medical Sciences under the reference code IR.QUMS.REC.1403.486. The entire research process was conducted in strict adherence to the principles outlined in the Declaration of Helsinki. The study’s purposes and procedures were thoroughly disclosed to all participants prior to their involvement. Written informed consent was obtained from every individual. Participants were explicitly assured that their participation was entirely voluntary, that all personal information would remain strictly confidential and anonymous, and that they retained the absolute right to withdraw from the study at any stage they wished without any academic or personal repercussions.

### Statistical analysis

Data were analyzed using IBM SPSS Statistics (version 27). Descriptive statistics, including means and standard deviations for continuous variables and frequencies with percentages for categorical variables, were calculated to summarize participant characteristics and study variables.

For the primary multivariable analysis, given the nested structure of the data (four moral disengagement subscales per student), we employed Generalized Estimating Equations (GEE) with a robust (Huber-White) covariance matrix estimator. This approach accounts for the within-subject correlation between the four subscale scores (behavioral distortion, agency distortion, outcome distortion, and dehumanization) while providing valid standard errors and hypothesis tests even in the presence of mild violations of the independence assumption. The model was fitted with a normal distribution and an identity link function, with all candidate predictor variables entered simultaneously using the forced entry method.

To select the optimal working correlation structure, we systematically compared three alternative structures: exchangeable (assuming equal correlation among all four subscales), unstructured (allowing unique correlations between each pair of subscales), and first-order autoregressive (AR1) (assuming correlations decrease with distance between measurements). Model fit was compared using the Quasi-likelihood under Independence Model Criterion (QIC) and the Corrected QIC (QICC), with smaller values indicating better fit. The exchangeable structure yielded the lowest QIC (7866.058) and QICC (7819.626) values, compared to AR1 (QIC = 7875.913; QICC = 7831.219) and unstructured (QIC = 34414.342; QICC = 32541.263). The unstructured structure demonstrated substantial overfitting, as evidenced by the excessively large QIC values, unstable parameter estimates, and a substantially inflated scale parameter. Therefore, the exchangeable working correlation structure was selected for the final model, providing a parsimonious and theoretically justified approach to modeling the shared nature of moral disengagement mechanisms.

The full model included all candidate predictors simultaneously and examined the interaction between subscale and emotional exhaustion to determine whether the predictive effect of emotional exhaustion differed across the four moral disengagement mechanisms. Model fit was assessed using the Omnibus test, the Quasi-likelihood under Independence Model Criterion (QIC), and the Corrected QIC (QICC). For each predictor retained in the final model, unstandardized coefficients (B) with their 95% confidence intervals (CIs), robust standard errors, Wald Chi-Square statistics, and standardized coefficients (β) were reported.

## Results

The final sample consisted of 203 nursing students with a mean age of 23.23 years (SD = 2.09). The majority were female (64.0%), single (92.1%), and residing at home (56.7%). Regarding academic characteristics, 50.2% were in the 7th semester and 49.8% in the 8th semester. Most students had voluntarily chosen nursing (71.9%) and expressed intention to work in the nursing field after graduation (70.0%). A detailed summary of participants’ demographic characteristics is presented in Table 1.

**Table 1:** Summary of participants’ demographic characteristics

		Mean (SD)/ N (%)
Age		23.23 (2.09)
Gender	Female	130 (64.0)
	Male	73 (36.0)
Semester	7th semester	102 (50.2)
	8th semester	101 (49.8)
Place of living	Home	115 (56.7)
	Dormitory	88 (43.3)
Marital status	Single	187 (92.1)
	Married	16 (7.9)
Voluntary working	Rarely	70 (34.5)
	Sometimes	99 (48.8)
	Often	34 (16.7)
How choose nursing subject	Voluntarily	146 (71.9)
	Obligatory	57 (28.1)
Intention to work in nursing field in future	No	30 (14.8)
	Not determined	31 (15.3)
	Yes	142 (70.0)

Table 2 presents the means, standard deviations, and Pearson correlation coefficients among all study variables. The mean total moral disengagement score was 44.87 (SD = 12.29), with agency distortion showing the highest mean score (M = 20.09, SD = 5.63), followed by behavioral distortion (M = 13.65, SD = 4.09), dehumanization (M = 5.61, SD = 2.24), and outcome distortion (M = 5.52, SD = 1.91). The mean emotional exhaustion score was 12.17 (SD = 3.98), and the mean psychological well-being score was 69.12 (SD = 9.73). All four moral disengagement subscales were strongly and positively intercorrelated (r ranging from 0.624 to 0.731, all *p* < 0.001), indicating substantial shared variance among the different mechanisms of moral disengagement. Emotional exhaustion showed significant positive correlations with all moral disengagement subscales (r ranging from 0.310 to 0.472, all *p* < 0.001), with the strongest association observed for agency distortion (*r* = 0.472, *p* < 0.001). Conversely, psychological well-being dimensions demonstrated significant negative correlations with moral disengagement. Specifically, personal growth showed moderate negative correlations with all four subscales and the total score (r ranging from − 0.257 to -0.315, all *p* < 0.001). Similarly, purpose in life was negatively correlated with all subscales (r ranging from − 0.181 to -0.294, *p* < 0.01). Self-acceptance also showed significant negative correlations with all subscales (r ranging from − 0.223 to -0.283, all *p* < 0.01). Environmental mastery was negatively correlated with outcome distortion (*r* = -0.152, *p* < 0.05) and dehumanization (*r* = -0.180, *p* < 0.05), while positive relations with others demonstrated significant negative correlations across all four subscales (r ranging from − 0.143 to -0.218, *p* < 0.05).

**Table 2:** Descriptive statistics and Pearson correlation coefficients among study variables

Variable	M	SD	1	2	3	4	5	6	7	8	9	10
1. Behavioral Distortion	13.65	4.09	—									
2. Agency Distortion	20.09	5.63	0.731***	—								
3. Outcome Distortion	5.52	1.91	0.652***	0.674***	—							
4. Dehumanization	5.61	2.24	0.728***	0.661***	0.624***	—						
5. Emotional Exhaustion	12.17	3.98	0.412***	0.472***	0.344***	0.310***	—					
6. Autonomy	9.08	2.03	-0.072	-0.061	0.010	-0.041	0.032	—				
7. Environmental Mastery	9.89	2.32	-0.098	-0.124	-0.152*	-0.180*	-0.163*	-0.050	—			
8. Personal Growth	12.31	2.11	-0.315***	-0.257***	-0.265***	-0.269***	-0.005	0.043	0.404***	—		
9. Positive Relations	12.53	3.06	-0.175*	-0.163*	-0.218***	-0.143*	-0.148*	-0.034	0.455***	0.354***	—	
10. Purpose in Life	12.76	2.87	-0.181**	-0.294***	-0.271***	-0.256***	-0.095	-0.079	0.325***	0.422***	0.176*	—
11. Self-Acceptance	12.56	3.13	-0.224**	-0.223**	-0.237***	-0.283***	-0.256***	-0.112	0.556***	0.334***	0.409***	0.465***

*M *Mean, *SD *Standard deviation

*p < 0.05. **p < 0.01. ***p < 0.001 (two-tailed)

Given the nested structure of the data (four subscales per student), we employed Generalized Estimating Equations (GEE) with robust standard errors to account for within-subject correlations. As detailed in the Methods section, the exchangeable working correlation structure was selected as the optimal structure following systematic comparison with AR1 and unstructured alternatives (exchangeable: QIC = 7866.058, QICC = 7819.626; AR1: QIC = 7875.913, QICC = 7831.219; unstructured: QIC = 34414.342, QICC = 32541.263). The final model demonstrated excellent fit (Omnibus test: χ² = 99.183, df = 9, *p* < 0.001; QIC = 7866.058; QICC = 7819.626). Table 3 presents the full GEE results. To ensure absolute clarity regarding the magnitude of effects, both unstandardized (B) and standardized (β) regression coefficients are reported where applicable.

**Table 3:** Generalized estimating equations (GEE) results predicting moral disengagement subscales

Effect	Predictor	B	Robust SE	95% CI	Wald χ²	*p*-value
Main Effects	Emotional Exhaustion	0.185	0.045	0.098–0.272	17.272	< 0.001
	Gender (Female vs. Male)	-1.444	0.360	-2.150 - (-0.738)	16.068	< 0.001
	Personal Growth	-0.281	0.101	-0.479 - (-0.084)	7.792	0.005
	Purpose in Life	-0.155	0.080	-0.312–0.003	3.704	0.054
	Choosing Nursing (Obligatory)	1.052	0.389	0.289–1.815	7.305	0.007
Interaction Effects	Subscale (Behavioral) × Emotional Exhaustion	0.249	0.048	0.156–0.342	27.316	< 0.001
	Subscale (Agency) × Emotional Exhaustion	0.494	0.073	0.350–0.638	45.205	< 0.001
	Subscale (Outcome) × Emotional Exhaustion	-0.009	0.033	-0.074–0.056	0.075	0.784
	Subscale (Dehumanization) × Emotional Exhaustion	0^*^	.	.	.	.

Model fit: QIC = 7866.058, QICC = 7819.626. Exchangeable working correlation structure. Robust standard errors (Huber-White estimator) used

*Reference category

*Main Effects*: The GEE analysis revealed that emotional exhaustion (Wald χ² = 52.823, *p* < 0.001) and gender (Wald χ² = 16.068, *p* < 0.001) were the strongest predictors of moral disengagement. Emotional exhaustion showed a significant positive effect (B = 0.185, 95% CI: 0.098 to 0.272; β = 0.461), while female gender was associated with significantly lower moral disengagement scores compared to males (B = -1.444, 95% CI: -2.150 to -0.738; β = -0.236). Among psychological well-being dimensions, only personal growth demonstrated a significant protective effect (B = -0.281, 95% CI: -0.479 to -0.084; β = -0.195; Wald χ² = 7.792, *p* = 0.005). Purpose in life showed a protective trend that approached but did not reach conventional statistical significance (B = -0.155, 95% CI: -0.312 to 0.003; β = -0.143; Wald χ² = 3.704, *p* = 0.054). Among control variables, obligatory choice of nursing was associated with significantly higher moral disengagement scores (B = 1.052, 95% CI: 0.289 to 1.815; Wald χ² = 7.305, *p* = 0.007). Other demographic variables, including age, semester, place of living, marital status, voluntary work, and intention to work in nursing, were not significant predictors (all *p* > 0.05). Similarly, other psychological well-being dimensions including autonomy, environmental mastery, positive relations with others, and self-acceptance did not show significant independent effects (all *p* > 0.05).

*Interaction Effects*: A significant interaction was observed between subscale and emotional exhaustion (Wald χ² = 52.356, df = 3, *p* < 0.001), indicating that the effect of emotional exhaustion varied significantly across different moral disengagement mechanisms. The strongest interaction effect was observed for agency distortion (B = 0.494, 95% CI: 0.350 to 0.638; Wald χ² = 45.205, *p* < 0.001), followed by behavioral distortion (B = 0.249, 95% CI: 0.156 to 0.342; Wald χ² = 27.316, *p* < 0.001). No significant interaction was found for outcome distortion (B = -0.009, *p* = 0.784), indicating that the effect of emotional exhaustion on outcome distortion was not significantly different from its effect on dehumanization (reference category).

## Discussion

This study sought to disentangle the concurrent predictive roles of emotional exhaustion and psychological well‑being in moral disengagement among final‑year nursing students. Its central finding was the identification of a competitive dual domain model in which emotional exhaustion, acting as an erosive force, and personal growth, functioning as a protective force, jointly predicted moral disengagement. Importantly, we observed a significant interaction between emotional exhaustion and moral disengagement subscales, indicating that the effect of emotional exhaustion was most pronounced for agency distortion—the mechanism involving displacement and diffusion of responsibility. The model demonstrated excellent fit confirming the robustness of this competitive framework. This substantial explanatory power underscores that moral disengagement within this population is not simply a fleeting reaction to situational pressures. Rather, it is deeply rooted in the erosion of psychological resources and the corresponding absence of robust internal psychological anchors. Framed within Hobfoll’s (1989) Conservation of Resources (COR) theory, this central finding suggests a clear dynamic: students who face the continuous drain of emotional energy progressively turn to low cost cognitive shortcuts, such as moral disengagement, to preserve what little psychological reserve they have left [24]. In direct contrast, the presence of well-established internal anchors, most notably a sustained sense of personal growth and a meaningful life direction, constitutes a protective shield that significantly reduces the perceived need for these maladaptive defensive mechanisms.

The overall level of moral disengagement among the students fell within a moderate range, a finding that aligns with reports from other Iranian nursing populations [36]. The most critical concern to emerge here is the documented presence of this phenomenon prior to the students’ formal entry into the nursing profession. This timing confirms that the seeds of moral disengagement are not sown exclusively by occupational conditions later on but are planted much earlier, during the preparatory phase of professional identity formation and through the gradual attrition of psychological resources across clinical education. This pattern is by no means culturally specific, as international evidence similarly confirms the presence of moral disengagement among practicing nurses [26, 37]. A legitimate risk therefore exists that these cognitive mechanisms become entrenched during this highly formative stage, a possibility that forcefully underscores the urgent need for screening programs and early preventive interventions throughout clinical internships, before such maladaptive patterns are fully consolidated.

Among the various dimensions of moral disengagement, agency distortion received the highest mean score. The primacy of this mechanism is theoretically well grounded in Bandura’s social cognitive theory, which identifies the “locus of agency” as one of four major centers of moral disengagement, operating specifically through the displacement and diffusion of responsibility [7]. Notably, this mechanism was also the most sensitive to emotional exhaustion, as evidenced by the significant interaction effect suggesting that resource depletion disproportionately amplifies the tendency to displace responsibility onto external factors. These intertwined mechanisms enable individuals to absolve themselves of ethical accountability by attributing fault to external factors, such as a strained healthcare system, chronic staffing shortages, or the orders of a supervising physician. In daily clinical practice, this cognitive shortcut is deeply grounded in the asymmetric power dynamics between clinical preceptors, staff nurses, and students. As students occupy the subordinate tier within this hierarchy, they navigate a double-edged sword: they may abdicate their moral responsibility by adopting the ‘just a student’ shield to satisfy demanding preceptors, or conversely, internalize the rigid clinical pressures imposed by staff nurses, leading to a complete surrender of their moral agency. This surrender functions not as a deliberate moral failure, but as a subtle psychological adaptation and defensive coping strategy to survive clinical socialization. This adaptive reaction mirrors other unconscious relational defense mechanisms in nursing such as countertransference tendencies, where clinicians deploy cognitive and emotional shields to cope with interpersonal stressors and prevent psychological depletion [38]. The inherently hierarchical and high stress nature of clinical environments provides an ideal substrate for their rapid activation. A recent qualitative study conducted among Iranian nurses powerfully corroborates this interpretation, identifying the “externalization of responsibility” as a pervasive justificatory mechanism; as one participant vividly stated, “If management were proper, maybe conditions would be different” [39]. Observing this identical pattern in our student sample demonstrates that the cognitive habit of self-exoneration takes root during undergraduate training. Nonetheless, the generalizability of this pattern across cultural contexts is not absolute. For instance, a study among ICU nurses in China identified “blame”, a mechanism pertaining to the victim locus, rather than agency distortion as the most prevalent mechanism [37]. This divergence plausibly reflects the high mortality environment of the ICU, where defensive mechanisms such as attributing fault to the patient or to unalterable circumstances dominate as a means of psychological self preservation. Such incongruence should not be regarded as a threat to our findings’ validity; rather, it presents an opportunity to emphasize the critical necessity of designing context sensitive interventions in ethics education.

The study further revealed that the level of emotional exhaustion among the nursing students was higher than average. This finding is in close alignment with results reported by Hong et al. (2024) among final year nursing students in China and is robustly reinforced by a comprehensive systematic review demonstrating that, globally, emotional exhaustion represents the single most prevalent form of burnout among nursing students [40]. This pattern has been replicated across diverse cultural and educational settings, including Spain, Chile, and South Africa [41–43]. Such high prevalence unequivocally confirms that the distinctive nature of clinical internships, marked by heavy workloads, continuous exposure to patient suffering, and hierarchical pressures, systematically depletes students’ emotional reserves.

The true significance of this finding, however, reaches far beyond its high prevalence alone. The most critical outcome was the identification of emotional exhaustion as the single strongest positive predictor of moral disengagement in the final GEE model. This finding converges strongly with a growing body of evidence from Iran and China that has established a direct link between affective depletion and the activation of moral disengagement [26, 36]. The mechanistic roots of this relationship can be elucidated by jointly applying the COR and “affect as information” frameworks. As established, moral self-regulation is an intensely resource-intensive process. When students are psychologically depleted, they become unable to sustain the high cognitive and emotional costs required for rigorous ethical deliberation. Consequently, negative affective states like exhaustion act as a cognitive “stop” signal-temporarily impairing high-level ethical reasoning and actively facilitating the use of justificatory shortcuts [24, 26]. This dual mechanism precisely explains why emotional exhaustion emerged as the most powerful driver of moral disengagement in our model: when psychological energy reserves are critically drained, the mind, in a fundamental effort to ensure psychological survival, diverts itself from the demanding path of ethical reflection and instead seeks refuge in cognitive shortcuts. This layered interpretation is also entirely consistent with the broader Stress Strain Outcome (SSO) framework, which posits that cumulative occupational and educational stressors generate psychological strain, which in turn produces negative behavioral outcomes such as moral disengagement [44].

In direct contrast, the average psychological well-being score of the students fell within a moderately high range, a finding consistent with studies by Du and Liu (2025) and Alaswad et al. (2025) [45, 46]. However, a study by Elbarbary et al. (2023) conducted in the wake of the COVID 19 pandemic reported substantially lower levels [28]. This discrepancy is most likely attributable to the distinct pressures of social isolation and virtual education experienced during that unique period, alongside potential differences in the measurement tools employed. Despite these contextual variations, the overall convergence of evidence signals that this psychological capital, even when present at moderate levels, remains fundamentally fragile and highly susceptible to erosion under sustained clinical pressures, a pattern that aligns closely with our findings on moral disengagement. Drawing on Bowlby’s (1982) attachment theory, psychological well-being can be compellingly conceptualized as an “internal secure base”, a foundational resource that equips adults to navigate complex ethical challenges without automatically resorting to destructive psychological mechanisms [47].

A particularly innovative and noteworthy finding was that the dimensions of psychological well-being did not exert an equal protective effect. Personal growth emerged as the only significant protective predictor among the six well-being dimensions. Personal growth, a state characterized by a continuous sense of development and the active realization of one’s potential, naturally subjects the individual to ongoing self‑evaluation and the revision of personal values. This continuous, self‑reflective cycle effectively blocks the pathway to justificatory cognitive shortcuts. Purpose in life showed a protective trend that approached but did not reach conventional statistical significance. This marginal attenuation in the GEE model, compared to the initial linear regression, may be attributed to the conservative nature of robust standard errors and the shared variance with personal growth. Nevertheless, the consistent negative direction of the coefficient supports its conceptual role as a protective factor, warranting further investigation in larger samples. Returning to Bowlby’s (1982) attachment theory [47], these two components can be viewed as the core constituents of a well developed “internal secure base.” Just as secure attachment in early childhood furnishes an external base from which to explore, personal growth and a strong sense of purpose create a stable psychological anchoring point that restrains individuals from resorting to maladaptive cognitive shortcuts when they encounter ethical conflicts. This finding is strongly corroborated by prior evidence, which suggests that meaningful protection against moral disengagement is achieved not through the broad, general enhancement of well-being, but rather through the targeted cultivation of specific components that together provide a durable internal value anchor [25, 37].

A novel contribution of this study is the significant interaction between emotional exhaustion and moral disengagement subscales, revealing that emotional exhaustion does not uniformly affect all mechanisms of moral disengagement. The strongest effect was observed for agency distortion, followed by behavioral distortion, while outcome distortion and dehumanization were not significantly more affected than the reference category. This differential pattern aligns with Bandura’s [7] theoretical conceptualization of agency distortion as a mechanism rooted in the externalization of responsibility. When students experience emotional depletion, they may be particularly inclined to displace responsibility onto external factors such as heavy workloads, staffing shortages, or physicians’ orders, as these attributions require minimal cognitive effort while effectively alleviating moral tension. Conversely, mechanisms such as dehumanization and outcome distortion may require more deliberate cognitive processing or may be activated through different pathways, making them relatively more resistant to transient affective states. This finding has important implications for intervention design. Specifically, educational programs should target agency distortion through structured case discussions that help students recognize and resist the tendency to externalize responsibility, particularly during periods of high emotional exhaustion.

Finally, male gender was significantly associated with higher moral disengagement scores. This finding is in full, uncontradicted agreement with the global literature on the topic [25, 26, 36]. This persistent gender difference can be compellingly interpreted through the dual lens of gender socialization theory and the ethics of care. Through sustained socialization into caregiving roles, women tend to cultivate a more robust ethical self concept and a heightened sensitivity to the interpersonal consequences of their actions, and they therefore demonstrate a measurably greater resistance to justificatory cognitive mechanisms [48]. This finding directly highlights the practical necessity of directing particular attention toward male nursing students in the design and delivery of professional ethics education programs.

### Implications for practice

The findings of this study carry implications for educational planners and policymakers within nursing education. First, the identification of emotional exhaustion as a predictor of moral disengagement serves as an indicator for clinical education administrators. It is recommended to implement structured programs for stress and emotional resource management throughout the clinical internship period. Specifically, incorporating evidence-based training modalities, such as structured emotional intelligence training and emotion regulation programs, into the nursing curriculum can provide students with necessary personal resources. Empirical research strongly supports this approach; recent evidence demonstrates that emotion regulation psychoeducation effectively helps nursing professionals manage emotional distress by significantly increasing adaptive cognitive reappraisal and reducing maladaptive emotional suppression [49]. Within the framework of COR theory, these competencies assist senior nursing students in managing clinical stressors, thereby buffering the transition from emotional depletion to moral disengagement. These modalities can be supplemented by facilitated group debriefing sessions and mindfulness skills training to mitigate the erosion of emotional resources.

Second, the findings indicate that protection against moral disengagement involves the cultivation of specific psychological dimensions, namely personal growth and a sense of purpose in life. A revision of the nursing curriculum is suggested to embed formal mentoring and professional coaching programs that assist students in defining their professional purpose and developing individualized growth plans. Mentorship programs, where experienced clinical nurses serve as professional role models, can contribute to the internalization of professional values and the reinforcement of students’ ethical standards.

Third, given the observed prevalence of the “agency distortion” mechanism, clinical instructors should incorporate structured case discussions into educational practice. These discussions can be designed to address the pattern of externalizing responsibility and to foster personal ethical accountability among students. Fourth, considering the variance observed among male students, the design and implementation of educational interventions that target ethical self-regulation in this subgroup is warranted.

### Strengths and limitations

This study has several strengths worth noting. Its primary contribution lies in the simultaneous examination of both erosive and protective psychological factors within a single predictive model, an approach that offers a more complete picture than studying either in isolation. The high response rate and the use of validated instruments with documented internal consistency support the reliability of the observed patterns. Furthermore, the application of Generalized Estimating Equations (GEE) with robust standard errors represents a methodological strength, as this approach appropriately accounts for the nested structure of the data (four moral disengagement subscales per student) and provides valid inference even in the presence of mild violations of the independence assumption.

These points should be considered alongside certain limitations. First, the cross-sectional design precludes causal inference; although we identified significant associations between emotional exhaustion, psychological well-being, and moral disengagement, the direction of these relationships cannot be definitively established. Second, the single-center sample limits the immediate generalizability of the findings to different institutional contexts, and future multicenter studies are needed to validate these results across diverse educational settings. Third, self-report measures, despite assurances of confidentiality, remain susceptible to social desirability bias, which may have influenced participants’ responses, particularly regarding sensitive topics such as moral disengagement and emotional exhaustion. Fourth, while we employed GEE with robust standard errors to address the mild positive autocorrelation observed in correlation matrix, the absence of a clustering variable (e.g., hospital ward or clinical instructor) prevented us from fully modeling the hierarchical structure of the data. Future studies should collect such contextual data to enable more sophisticated multilevel analyses. Fifth, a conceptual nuance arises from applying classical inferential statistics within a near-complete census framework (91.44% response rate). While frequentist hypothesis testing formally assumes random sampling from an infinite universe, our analytical approach treats this cohort as a cross-sectional realization of an underlying, longitudinal data-generating mechanism (a superpopulation framework). This approach justifies the use of *P*-values and confidence intervals to examine structural relationships, making the findings relevant for future student cohorts within similar clinical learning environments. Finally, although GEE is robust to misspecification of the correlation structure, the exchangeable working correlation assumed equal correlations among all four subscales, which may not fully capture the complex pattern of relationships between specific moral disengagement mechanisms. Future longitudinal and multicenter investigations utilizing mixed-methods designs are warranted to further validate and extend these findings, with particular attention to collecting clustering variables and employing more flexible correlation structures. Finally, while we systematically compared exchangeable, AR1, and unstructured working correlation structures and selected the exchangeable structure based on QIC values and model stability, alternative correlation structures might be considered in future studies with larger samples or different data structures.

## Conclusion

This cross-sectional study provides evidence that moral disengagement is a detectable phenomenon among nursing students during their clinical internship period, with agency distortion, described as the tendency to displace or diffuse responsibility onto external factors, emerging as both the most prevalent mechanism and the one most closely associated with emotional exhaustion. The findings suggest that emotional exhaustion may act as a primary contributor to moral disengagement, while personal growth (and, to a lesser extent, a sense of purpose in life) appears to serve as a protective factor. A notable observation was the differential vulnerability of moral disengagement mechanisms to emotional depletion, with agency distortion showing the strongest association. These findings, however, should be interpreted with caution given the cross-sectional nature of the study, which precludes causal inferences. Nevertheless, they highlight the potential value of educational interventions that address emotional exhaustion while fostering personal growth and a coherent sense of purpose. Particular attention might be directed toward helping students recognize and resist the tendency to externalize responsibility, especially during periods of high clinical stress. Future longitudinal and multicenter research is needed to establish the directional nature of these relationships and to evaluate the effectiveness of preventive interventions designed to promote ethical resilience in nursing students.

## Data Availability

The datasets used and analyzed during the current study are available from the corresponding author on reasonable request.
